# Effects of hyaluronic acid on bleeding following third molar extraction

**DOI:** 10.1590/1678-77572015-0187

**Published:** 2017

**Authors:** Gokhan GOCMEN, Sertac AKTOP, Burcin TÜZÜNER, Bahar GOKER, Aysen YARAT

**Affiliations:** 1 Marmara University, Faculty of Dentistry, Department of Oral and Maxillofacial Surgery, İstanbul, Turkey.; 2 Marmara University, Faculty of Dentistry, Department of Biochemistry, Istanbul, Turkey.; 3 Marmara University, Faculty of Pharmacy, Department of Biochemistry, Istanbul, Turkey.

**Keywords:** Hyaluronic acid, Bleeding, Extraction

## Abstract

**Objective:**

To explore the effects of hyaluronic acid (HA) on bleeding and associated outcomes after third molar extraction.

**Methods:**

Forty patients who had undergone molar extraction were randomly divided into two groups; 0.8% (w/v) HA was applied to the HA group (n=20) whereas a control group (n=20) was not treated. Salivary and gingival tissue factor (TF) levels, bleeding time, maximum interincisal opening (MIO), pain scored on a visual analog scale (VAS), and the swelling extent were compared between the two groups.

**Results:**

HA did not significantly affect gingival TF levels. Salivary TF levels increased significantly 1 week after HA application but not in the control group. Neither the VAS pain level nor MIO differed significantly between the two groups. The swelling extent on day 3 and the bleeding time were greater in the HA group than in the control group.

**Conclusions:**

Local injection of HA at 0.8% prolonged the bleeding time, and increased hemorrhage and swelling in the early postoperative period after third molar extractions.

## Introduction

Hyaluronic acid (HA) exerts an anti-inflammatory effect during oral wound-healing, and is commonly applied after tooth extraction. Generally, previous studies about the subject have focused on tissue inflammatory responses after extrinsic HA application. It has been hypothesized that HA of appropriate consistency increases cell motility^1,13^.

Tissue factor (TF) is best known as the primary cellular initiator of blood coagulation. TF levels vary as the need for hemostatic protection changes^4,10^. The initial effect of HA on tissue hemostasis may reflect changes in tissue TF levels. The bleeding time may also be an objective measure of HA activity. Swelling, pain scored on a visual analog scale (VAS), and maximum inter-incisal opening (MIO) may also reflect the hemorrhage extent.

The anti-inflammatory efficacy of HA, and the positive outcomes of HA treatment, may cause researchers to overlook potential side effects. Considering its probable efficiency on overall healing process, hemostatic effects of HA should also be considered. Our hypothesis was that HA might modulate hemostasis and bleeding. Additionally its therapeutic effect and relationship with side effects can also compromise its use in surgical procedures. The purpose of this study was to measure the ability of high-molecular weight HA to induce hemostasis after mandibular third molar (M3) extraction. The primary measures were the TF level and bleeding extent; the VAS pain score, MIO, and swelling extent served as secondary clinical measures.

## Materials and methods

### Patients and Methods:

**Figure 1:**
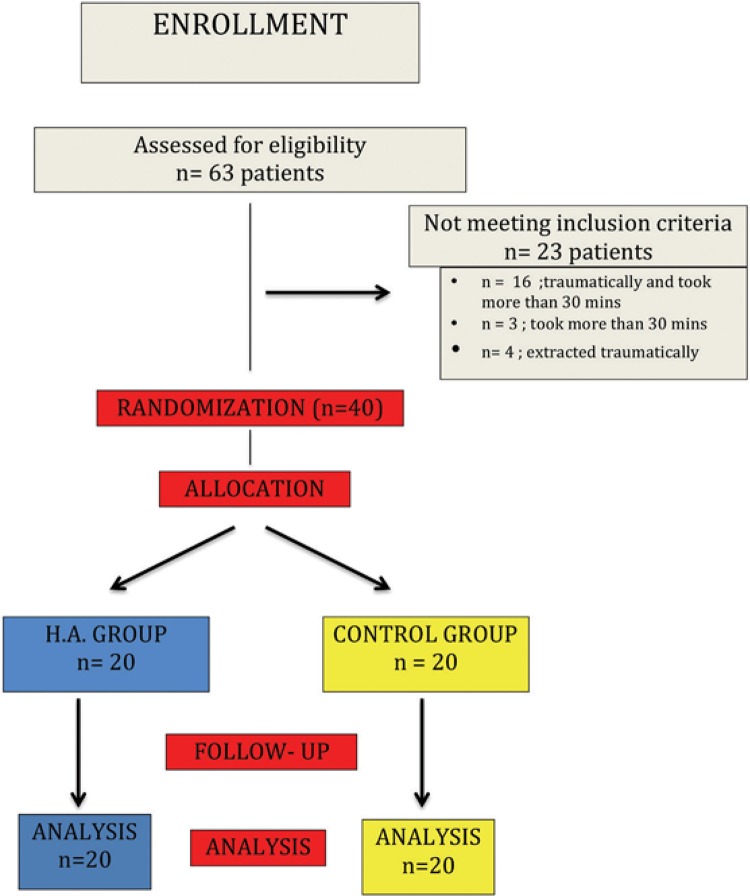
Study flowchart

An a priori power calculation indicated that a sample size of 18 patients was required for each group. This comparative, prospective randomized study included 40 patients treated in the Department of Oral and Maxillofacial Surgery, at the Faculty of Dentistry, Marmara University, Istanbul, Turkey. Approval was obtained from the appropriate Ethics Committee (approval no. 2011-1). Written informed consent was obtained from all patients. Eligibility criteria to participate in the study required patients to have vertical half-impacted M3 without bony retention, to be non-smoker and classified as ASA I according to the guidelines of the American Society of Anesthesiology. Exclusion criteria included the use of any antibiotic or anti-inflammatory medication within the 2 weeks prior to surgery, and/or any pathological or inflammatory condition in the impacted tooth area (n: 65). Additionally, after submission to the surgery, if the extraction was traumatic, patients were excluded from the study – i.e., the tooth may require or underwent separation, fracture during extraction, removal surrounding bony support or full thickness mucoperiosteal flap elevation, and/or total extraction time (time passed during local anesthetic administration and anesthesia onset were excluded, only extraction period was accounted from start to end) took more than 30 minutes. Both groups contained elective patients who had undergone unilateral mandibular M3 extraction (Figure 1).

The primary predictor, HA application, was coded as a binary variable. Half of the patients were randomly assigned to receive a local HA gel (0.8% [w/v]; Gengigel, Ricerfarma, Italy) following tooth removal (n=20)^5,6^. A 0.2 ml HA gel was applied immediately after M3 removal to the edge of the extraction socket. The control group was not treated.

Before each surgical procedure, MIO was recorded (in mm) and the mouth was rinsed with distilled water. Saliva samples (2 mL) were collected by spitting into a funnel while in the resting position. Teeth were removed under local anesthesia (2% [w/v] articaine HCl with 1:100,000 [v/v] epinephrine HCl; Ultracaine D-S Forte; Aventis, Bridgewater, NJ, USA). Tissue samples approximately 1 mm^3^ in volume were collected immediately after extraction (T0) from the edge of the buccal wound. Wound closures were made with 3.0 silk sutures. Bleeding time(s) after wound closure was noted. Saliva samples (2 mL) were collected again exactly 1 h (T1) following extraction. Bleeding time and TF levels (salivary and gingival) served as the primary outcome variables. Postoperatively, amoxicillin (1,000 mg every 12 h for 1 week) and ibuprofen (400 mg every 6 h for 48 h) were prescribed for all patients. No steroid was prescribed for any patient. All patients were instructed to rinse their mouths twice daily with 0.2% (w/v) chlorhexidine.

Swelling, the pain score on a VAS, and MIO served as secondary outcome variables. All patients were followed-up on postoperative days 3 and 7, regarding assessment of swelling, post-surgical pain, and MIO. To measure swelling, orotragal and mentotragal distances (thus from the most inferior region of the tragus to the oral commissure, and to the soft tissue margin, respectively) on the operated side were noted^11^. VAS pain scores were recorded 1 h, and 3 and 7 days, after surgery. At 1 week (T2), the mouth was rinsed with distilled water and 2-mL saliva samples collected once more as described above. The sutured regions were locally anesthetized and tissue samples were obtained from sites very close to the initial sampling sites.

All tissue samples were initially stored at −94°C and transferred to −24°C 2 days before the performance of tests. On the day of TF measurements, thawed tissue samples were washed with saline, all veins and blood carefully removed, the samples dried on filter paper, and wet weights recorded. The tissue samples were then homogenized in saline and TF activities in the homogenates, and saliva samples measured using Quick’s one-stage method employing pooled plasma from healthy participants. Each TF activity measurement was performed by mixing 0.1 mL of tissue homogenate or saliva with 0.1 mL 0.02 M CaCl_2_, and the clotting reaction started by the addition of 0.1 mL of plasma^9^. All reagents were brought to the reaction temperature (37°C) prior to admixture. As the clotting time is inversely proportional to the TF activity level, lengthening of clotting time is a manifestation of reduced TF activity.

All statistical analysis was performed using the SPSS for Windows software package (ver. 12.0; SPSS Inc., Chicago, IL, USA). Descriptive statistics were computed for all variables. The t-test, and Mann-Whitney U test were used to assess differences between the HA and control groups. A p value <0.05 was considered to reflect statistical significance.

## Results

The mean patient age was 24.8 years (range: 18-35 years). Comparison of the gingival TF between groups was not statistically significant (Table 1). Application of HA did not make a statistically significant difference with TF when compared to the control group’s mean values.

**Table 1:** Comparison of gingival Tissue Factor (TF) between control and hyaluronic acid groups

TF	Control Group	H.A. Group	
	Mean ± SD	Mean ± SD	
Pre-op	102.15±31	89.80±24.88	
Post-op (1 week)	63.10±16.2	54.15±14.03	
+p	<0.0001	<0.0001	
Median	−36.3	−39.3	*p
(Min ; Max)	(−69.3 ; 51.6)	(−70.3 ; 21.8)	
			0.758

+: t test

*: Mann-Whitney U test

In the control group, there was no statistically significant difference of TF in 3 different measurement means. Salivary TF was significantly lesser after 1 week in HA group in One-Way ANOVA for Repeated Measurement test (p<0,05). In this group, binary variables were compared with LSD (Least Significant Difference) test. In which, mean of T2 was less then both T0 and T1. LSD test showed no significant difference between T0 and T1 (Table 2).

**Table 2:** Comparison of salivary Tissue Factor (TF) between control and hyaluronic acid groups

TF	Control Group	H.A. Group	
	Mean ± SD	Mean ± SD<	
Pre-op	87.1±26.5	122.3±48	
Post-op (1 hour)	97.55±37.48	114.20±31.48	
Post-op (1 week)	84.8±29.2	68.6±22.98	
+p	0.081	<.0001	
(Pre-op vs Post-op 1 hour)			*p
Median	−2	−32.21	0.001
(Min ; Max)	(-40.96 ; 59.72)	(−77.27; 48.00)	
(Pre-op vs Post-op 1 week)			
Median	−14.63	−42.81	0.002
(Min ; Max)	(−51.19 ; 51.32)	(−68.42 ; .00)	

+: Repeated measures ANOVA

*: Mann-Whitney U test

The mean ± SD of bleeding time in HA group showed statistically significant difference from control group. Bleeding time was longer in HA group than in the control group. (7.17±١.٣٦ versus ٥.٦٤±١.٤٦ minutes, p<٠.05 ). Comparison of pre-op, 1 hour, 3^rd^day and 7^th^ day MIO and VAS were not statistically significant between groups. Swelling was not significantly different at pre-op, 1 hour and 7^th^ day measurements (p<0.05). However 3rd day outcomes of orotragus and mentotragus measurements showed more swelling in the HA group (p<0.05)(Table 3).

**Table 3:** Comparison of VAS, MIO and swelling between control and hyaluronic acid groups

	MIO			Swelling						VAS		
	Mean ± (SD)			Mean ± (SD)						Mean± (SD)		
				HA		Control		p		HA	Control	
	HA Group (mm)	Control Group (mm)	p	Group (mm)		Group (mm)				Group	Group	p
				O.T	M.T.	O.T.	M.T.	O.T.	M.T.			
Pre-op	39.4 ±(2.3)	40.1 ±(2.2)	>0.05	104.36 ±(3.32)	140.4 ±(4.6)	105.2 ±(3.4)	140.18 ±(4.3)	>0.05	>0.05	0	0	
1 hour	39.2 ±(2.5)	39.4 ±(2.3)	>0.05	105.8 ±(3.76)	142.2 ±(4.1)	106.23 ±(2.8)	140.7 ±(3.8)	>0.05	>0.05	1.2 ±(0.4)	1.4 ±(0.5)	>0.05
3^th^ day	35.6 ±(1.6)	34.82 ±(1.8)	>0.05	113 ±(2.66)	148.3 ±(2.48)	110 ±(2.1)	144.6 ±(2.6)	<0.05	<0.05	4.3 ±(1.6)	4.5 ±(1.2)	>0.05
7^th^ day	38.6 ±(2.2)	37.8 ±(2.5)	>0.05	106.5 ±(2.8)	141.48 ±(4.4)	107.8 ±(3.8)	141.2 ±(3.2)	>0.05	>0.05	3.1 ±(1.4)	3.4 ±(1.83)	>0.05

## Discussion

We explored whether HA affected hemostasis after M3 extraction. We used the TF level and bleeding time as primary outcomes and VAS-measured pain, MIO, and swelling as secondary outcomes. To the best of our knowledge, the effect of high-molecular weight HA on hemostasis after M3 extraction has not previously been examined.

HA inhibits platelet aggregation and adhesion and, at high concentrations, prolongs bleeding times. As HA exerts antithrombotic effects, the material is used to coat endovascular devices^15^. HA plays two very important roles during wound-healing. First, it creates a temporary structure during the early stages of healing. Second, and most importantly, it triggers cell proliferation and migration^14^. Therefore, HA is often used to aid oral wound-healing and increases leukocyte diapedesis and fibroblast proliferation^12^. However, although HA aids wound-healing, this may be associated with increased bleeding (an antithrombotic effect)^2,15^. We measured the TF levels, the blood coagulation cascade initiator, to explore the relationship between HA application and bleeding.

TF is often overexpressed after wounding, trauma, or surgery. TF-induced hypercoagulability encourages wound-healing^3^. However, we found that HA did not increase gingival TF activity. The salivary TF level was significantly higher in the HA group than in the controls at 1 week after operation, when we expected a reduction. This may be associated with prolongation of bleeding in the HA group. HA increased salivary but not gingival TF levels, perhaps because physiological changes in the salivary glands and gingiva differ.

HA is a natural component of the extracellular matrix, enabling a structural framework, helping hydration, and thus creating a non-immunogenic environment that assists regeneration^6^. HA might affect the clinical outcomes of inflammation, supporting wound-healing and thus having clinical applications. Koray, et al.^8^ (2014) reported less pain and a reduced MIO after M3 extraction in patients treated with HA. Hanci and Altun^7^ (2015) reported post-tonsillectomy pain relief and increased wound-healing upon HA application. HA also controlled gastrointestinal bleeding after failed endoscopic therapy^9^. In our study, we measured early clinical outcomes and the extent of bleeding immediately after extraction. HA usage was associated with more swelling and more prolonged bleeding. However, the bleeding effect was short-term (2 or 3 days); outcomes became similar later in both groups.

## Conclusion

Local injection of HA at 0.8% prolonged the bleeding time, increased hemorrhage and swelling in the early postoperative period after M3 extractions. However, hemostasis and hemorrhage to induce wound healing are complex mechanisms and involve numerous parameters. Further work on other coagulation factors, measuring other clotting parameters in more patients, is required.

## References

[1] Aya KL, Stern R (2014). Hyaluronan in wound healing: rediscovering a major player. Wound Repair Regen.

[2] Basora JF, Fernandez R, Gonzalez M, Adorno J (2014). A case of diffuse alveolar hemorrhage associated with hyaluronic acid dermal fillers. Am J Case Rep.

[3] Chen J, Kasper M, Heck T, Nakagawa K, Humpert PM, Bai L (2005). Tissue factor as a link between wounding and tissue repair. Diabetes.

[4] Emekli-Alturfan E, Kasikci E, Alturfan AA, Pisiriciler R, Yarat A (2009). Effect of sample storage on stability of salivary glutathione, lipid peroxidation levels, and tissue factor activity. J Clin Lab Anal.

[5] Gocmen G, Gonul O, Oktay NS, Yarat A, Goker K (2015). The antioxidant and anti-inflammatory efficiency of hyaluronic acid after third molar extraction. J Craniomaxillofac Surg.

[6] Gontiya G, Galgali SR (2012). Effect of hyaluronan on periodontitis: a clinical and histological study. J Indian Soc Periodontol.

[7] Hanci D, Altun H (2015). Effectiveness of hyaluronic acid in post-tonsillectomy pain relief and wound healing: a prospective, double-blind, controlled clinical study. Int J Pediatr Otorhinolaryngol.

[8] Koray M, Ofluoglu D, Onal EA, Ozgul M, Ersev H, Yaltirik M (2014). Efficacy of hyaluronic acid spray on swelling, pain, and trismus after surgical extraction of impacted mandibular third molars. Int J Oral Maxillofac Surg.

[9] Lee JW, Kim HH (2014). Hyaluronic acid solution injection for upper and lower gastrointestinal bleeding after failed conventional endoscopic therapy. Dig Endosc.

[10] Mackman N (2004). Role of tissue factor in hemostasis, thrombosis, and vascular development. Arterioscler Thromb Vasc Biol.

[11] Mahmoud Hashemi H, Mohammadi F, Hasheminasab M, Mahmoud Hashemi A, Zahraei S, Mahmoud Hashemi T (2015). Effect of low-concentration povidone iodine on postoperative complications after third molar surgery: a pilot split-mouth study. J Oral Maxillofac Surg.

[12] Mesa FL, Aneiros J, Cabrera A, Bravo M, Caballero T, Revelles F (2002). Antiproliferative effect of topic hyaluronic acid gel. Study in gingival biopsies of patients with periodontal disease. Histol Histopathol.

[13] Neuman MG, Nanau RM, Oruna-Sanchez L, Coto G (2015). Hyaluronic acid and wound healing. J Pharm Pharm Sci.

[14] Tammi MI, Day AJ, Turley EA (2002). Hyaluronan and homeostasis: a balancing act. J Biol Chem.

[15] Verheye S, Markou CP, Salame MY, Wan B, King SB, Robinson KA (2000). Reduced thrombus formation by hyaluronic acid coating of endovascular devices. Arterioscler Thromb Vasc Biol.

